# Interactome of the negative regulator of nuclear import BRCA1-binding protein 2

**DOI:** 10.1038/srep09459

**Published:** 2015-03-30

**Authors:** Shadma Fatima, Kylie M. Wagstaff, Kate L. Loveland, David A. Jans

**Affiliations:** 1 Department.of Biochemistry & Molecular Biology Monash University, Clayton, Victoria, Australia; 2 Department of Anatomy and Developmental Biology, Monash University, Clayton, Victoria, Australia

## Abstract

Although the negative regulator of nuclear import (NRNI) BRCA1 binding protein 2 (BRAP2) is highly expressed in testis, its role is largely unknown. Here we address this question by documenting the BRAP2 interactome from human testis, using the yeast 2-hybrid system to identify BRAP2-interacting proteins with roles in diverse cellular processes, including regulation of the actin cytoskeleton, ubiquitinylation, cell cycle/apoptosis and transcription. Interaction with BRAP2 in adult mouse testis with three of these, PH domain and leucine rich repeat protein phosphatase 1 (PHLPP1), A-Kinase anchor protein (AKAP3) and DNA methyl transferase 1 (DNMT1), was confirmed by coimmunoprecipitation assays. BRAP2's ability to inhibit PHLPP1 and DNMT1 nuclear localisation was also confirmed by quantitative confocal microscopy. Importantly, the physiological relevance thereof was implied by the cytoplasmic localisation of PHLPP1, AKAP3 and DNMT1 in pachytene spermatocytes/round spermatids where BRAP2 is present at high levels, and nuclear localisation of PHLPP1 and DNMT1 in spermatogonia concomitant with lower levels of BRAP2. Interestingly, BRAP2 was also present in murine spermatozoa, in part colocalised with AKAP3. Together the results indicate for the first time that BRAP2 may play an important NRNI role in germ cells of the testis, with an additional, scaffold/structural role in mature spermatozoa.

Regulated nucleocytoplasmic trafficking of macromolecules is critical to a range of eukaryotic cellular processes, including oncogenesis, differentiation and development^1^,^2^,^3^,^4^. An example is mammalian spermatogenesis, by which an ordered, sequential series of germ cell type changes lead to a mature spermatozoan able to fertilise a mature ovum, where the regulation of nuclear transport in response to cellular signals is a key driver^5^. One mechanism by which nucleocytoplasmic transport can be regulated is through negative regulators of nuclear import (NRNIs), of which the BRCA1 binding protein 2 (BRAP2) is an example that acts by binding to the nuclear localisation signal (NLS) of cargo proteins such as BRCA1, cyclin-dependent kinase inhibitor 1 (p21) and viral proteins including the SV40 large tumour antigen (T-ag) and human papilloma virus proteins^2^,^6^,^7^, and retaining them in the cytoplasm. Dysregulation of BRAP2 is also linked to cancers^8^,^9^,^10^,^11^ and inflammatory dysfunction of cardiovascular system in humans^12^, underlining its importance as a cell regulator.

Although, highly expressed in testis^13^, the specific role of BRAP2 and the target proteins it may bind is largely unknown. To address this directly, we performed a yeast-2-hybrid (Y2H) screen using an adult human testis cDNA library, to determine the BRAP2 testicular interactome (see Ref. 14). The interactome includes proteins involved in regulation of the actin cytoskeleton, ubiquitinylation, cell cycle/apoptosis and transcription. We validate interaction with three of these; the PH domain and leucine rich repeat protein phosphatase 1 (PHLPP1), DNA methyl transferase 1 (DNMT1) and a testis specific A-Kinase anchor protein 3 (AKAP3), as targets of BRAP2 action for the first time, underlining BRAP2's role not only as an NRNI, but as a component within mature spermatozoa that may fulfil a structural or scaffold role.

## Results

### The BRAP2 interactome in testis

**Figure 1:**
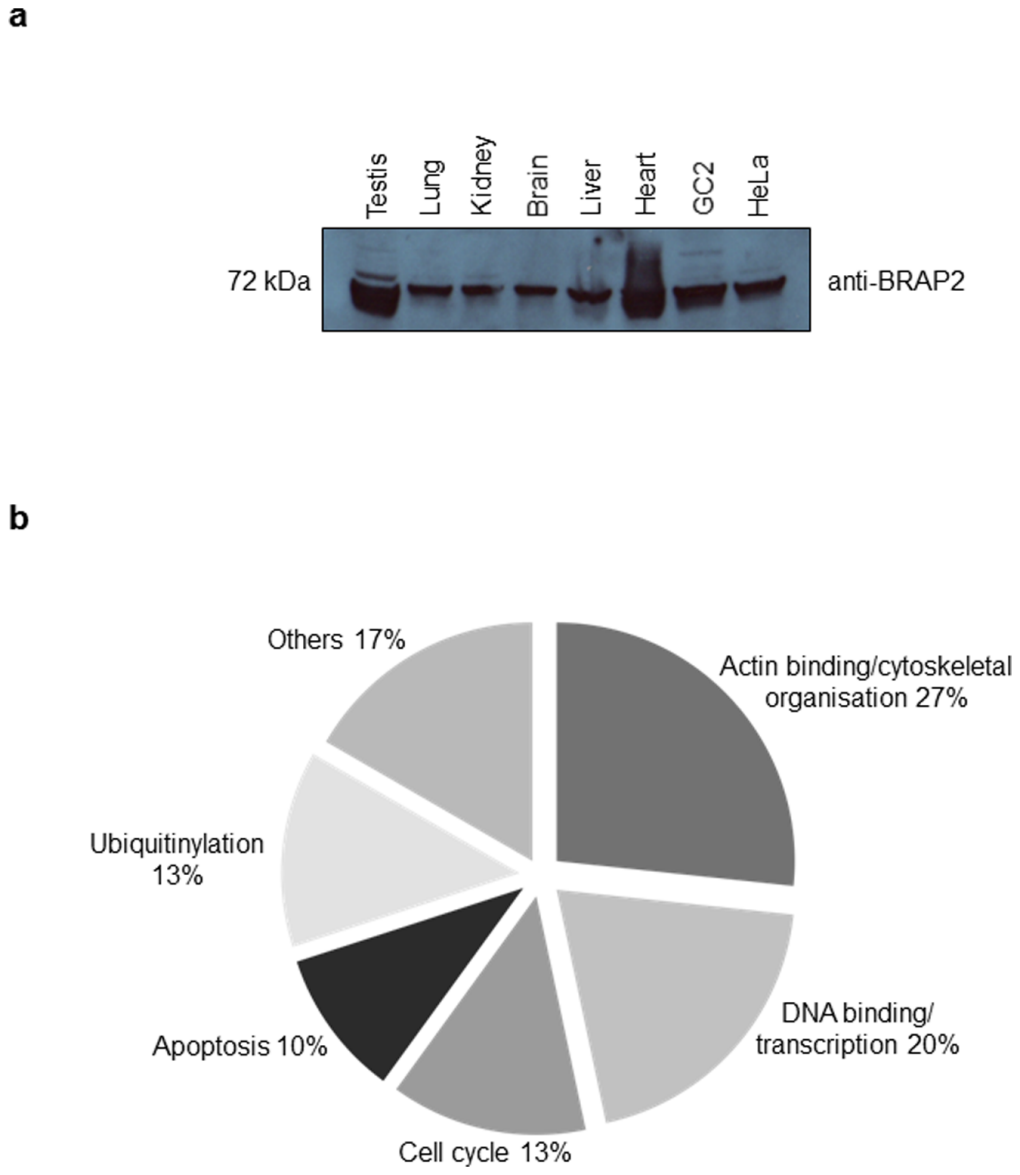
BRAP2 and its binding partners are highly expressed in testis. (a) Western analysis for expression of BRAP2 protein in normal mouse tissues and in HeLa/GC2 cell lines performed as per the methods section. (b) Classification of potential BRAP2 binding proteins identified in Y2H screen according to broad functions as per data mining.

Although BRAP2 is highly expressed in the mammalian testis (Fig. 1a; see Ref. 13), very little is known about its specific role. To address this question, we performed a large scale yeast-2-hybrid (Y2H) screen to identify binding partners of BRAP2 from a human testis cDNA library (see also Ref. 14). We identified a total of 27 proteins (see Table 1), which constitute the first definitive insight into BRAP2 interactome. The interactors could be categorised as playing roles in actin binding/cytoskeletal organisation, DNA/RNA binding/transcriptional regulation, cell cycle/proliferation, ubiquitin signalling pathways and cell apoptosis (see Fig. 1b). The identification of UBB and UBC as BRAP2 interactors, in agreement with previous studies^15^,^16^,^17^ is consistent with BRAP2's E3 Ubiquitin ligase activity. Of the novel protein binding partners identified, tumour suppressor protein PH domain and leucine rich repeat protein phosphatase 1 (PHLPP1), a testis specific A-Kinase anchor protein 3 (AKAP3), and DNA methyl transferase 1 (DNMT1)^18^,^19^,^20^ were selected for validation.

### BRAP2 interacts with PHLPP1, DNMT1 and AKAP3 in mouse testis

**Figure 2:**
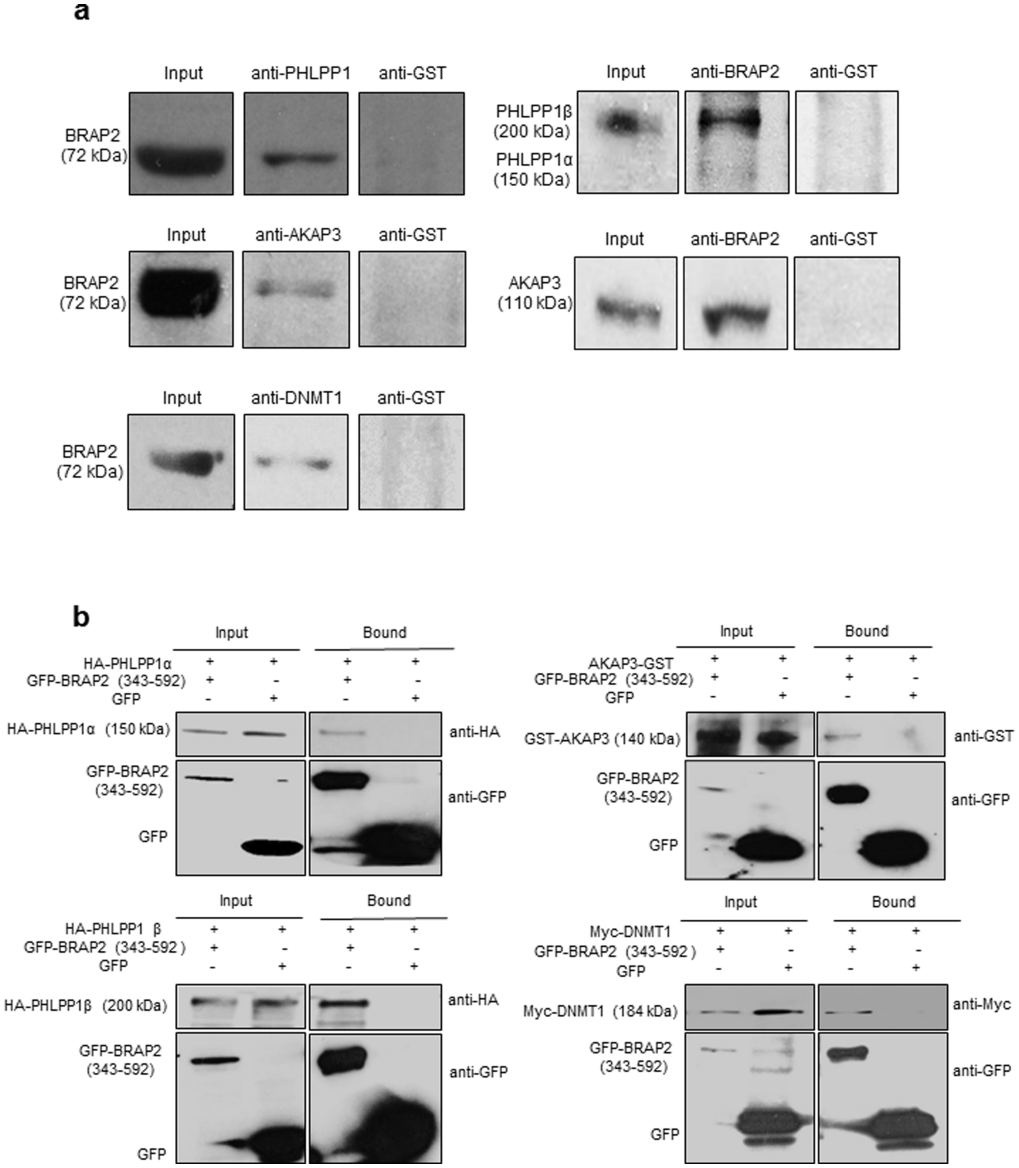
BRAP2 interacts with PHLPP1, AKAP3 and DNMT1 in mouse testis. (a) Coimmunoprecipitation (IP) experiments were performed as described in methods from adult mouse testis lysate using anti-PHLPP1, AKAP3, DNMT1 or GST antibodies and subjected to Western analysis using anti-BRAP2 (left) where as anti-BRAP2 antibody was used for IP from the same lysates and subjected to Western analysis using anti-PHLPP1, AKAP3 or anti-GST antibody (right) (b) GFP pull downs were performed from Hek293T cells co-transfected to express either GFP-BRAP2 (343-592), or GFP and HA-PHLPP1 α, or HA-PHLPP1 β or GST-AKAP3 or Myc-DNMT1 prior to Western analysis of GFP-trap-precipitated (bound) fractions using specific antibodies to GFP.

As a first step, coimmunoprecipitation was performed from adult mouse testis lysate using antibodies specific to PHLPP1, AKAP3 or DNMT1 (Fig. 2a, left) or anti-GST antibody as a negative control. Western analysis indicated that endogenous BRAP2 co-immunoprecipitated with specific antibodies to PHLPP1, AKAP3 or DNMT1, but not with the control antibody, indicating that BRAP2 interacts with these proteins in adult mouse testis. As confirmation, the coimmunoprecipitation was performed using antibody specific to BRAP2, Western analysis indicating that endogenous PHLPP1 (largely the alternatively spliced β-isoform) and AKAP3 could be coimmunoprecipitated with antibodies to BRAP2, but not with control antibody (Fig. 2a, right). Interestingly, BRAP2 interacts with both the α- and the alternatively spliced β-isoform of PHLPP1 (see also Fig. 2b), which selectively dephosphorylate specific AKT isoforms, promoting apoptosis and reducing cell proliferation^21^. Binding of BRAP2 to both PHLPP1 isoforms may modulate the synergistic action of the PHLPP1 isoforms, providing a strong regulatory mechanism to balance cell proliferation with apoptosis.

In the case of DNMT1, bands lower than that of full length DNMT1 were obtained (not shown) implying degradation; accordingly pull downs were also performed using Hek293T cells co-transfected to transiently express GFP-BRAP2 (343-592) or GFP alone along with Myc-tagged DNMT1, as well as HA-tagged PHLPP1 α- or β-isoforms and GST-tagged AKAP3. All four ectopically expressed proteins were pulled down with GFP-BRAP2 (343-592), but not with GFP alone (Fig. 2b), supporting the results obtained for testis lysates for DNMT1, AKAP3, and both isoforms of PHLPP1.

### BRAP2 can acts as an NRNI for PHLPP1 and DNMT1

**Figure 3:**
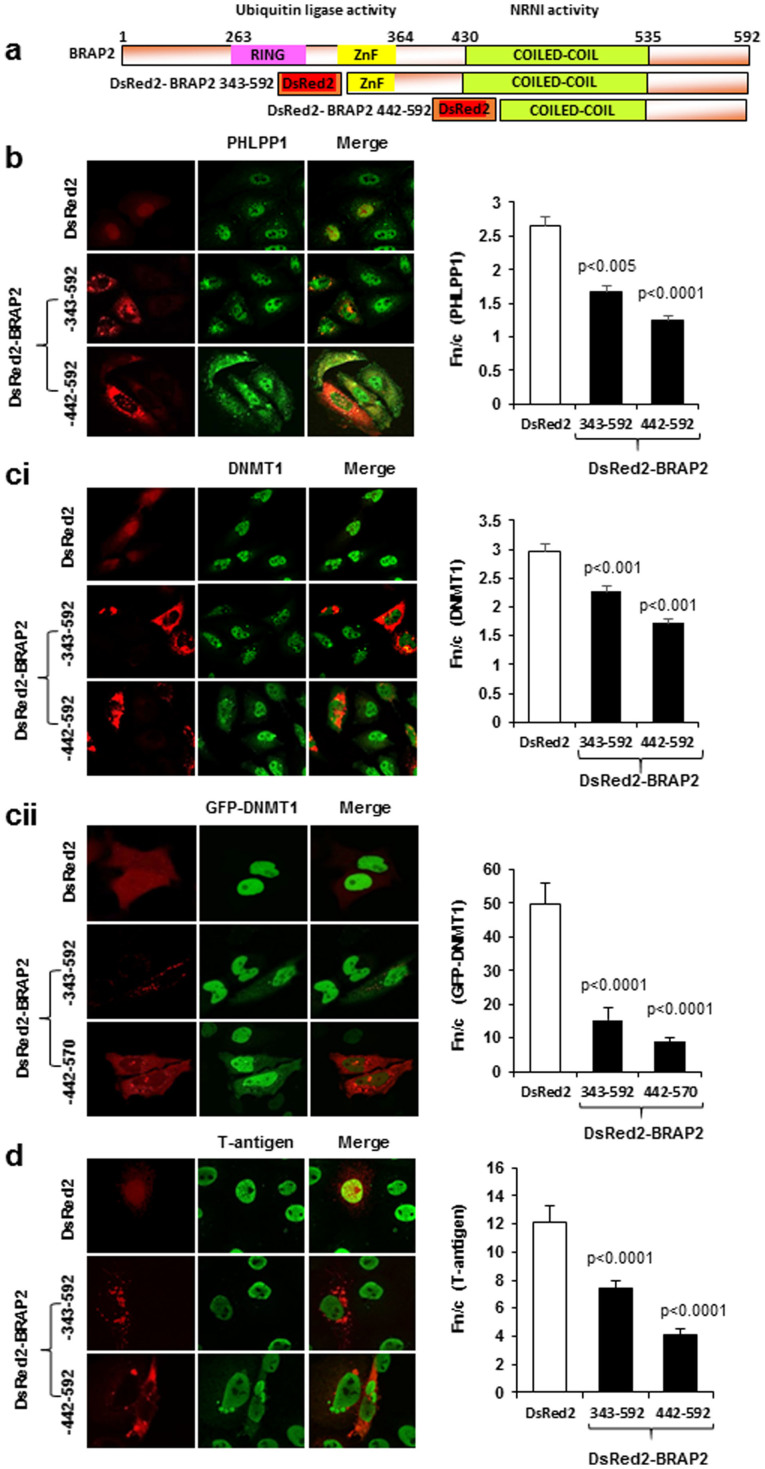
BRAP2 can acts as cytoplasmic retention factor for PHLPP1 and DNMT1. (a) Schematic representation of domain structure of BRAP2 and DsRed2 fused BRAP2 343–592 and 442–592. Amino acid residue numbers are indicated (top) Ring domain = 263–303; Zinc-finger ubiquitin binding domain (ZnF UBP) = 315–364; Coiled coil domain = 430–535. (b) CLSM images of HeLa cells transfected to express DsRed2 vector or DsRed2 fused BRAP2 constructs (as indicated) followed by fixation 20 hours post transfection and immunostained for endogenous PHLPP1 (left panels). Quantitative analysis on digitized CLSM images (right panel) to determine the Fn/c ratio (nuclear to cytoplasmic fluorescence ratio, above background) for endogenous PHLPP1 protein in presence and absence of indicated DsRed2 fused proteins. Values represent the mean +/− SEM (n > 30), with p values (Student's t test) shown where there were significant differences between values in absence or presence of the BRAP2 constructs (right). (ci) As per (b), CLSM images of HeLa cells immunostained with DNMT1 antibody in presence or absence of ectopically expressed DsRed2 fused BRAP2 constructs (as indicated on panels) or DsRed2 vector (left) together with quantitative analysis (right panel). (cii) As per (b), CLSM images of live HeLa cells co-transfected for ectopic expression of GFP-DNMT1 with DsRed2 or DsRed2 fused BRAP2 343–592 and 442–570 post 20–24 hours (left), together with quantitative analysis (right panel). (d) As per (b), CLSM images of Cos7 cells immunostained with T-ag antibody in presence or absence of ectopically expressed DsRed2 fused BRAP2 constructs (as indicated on panels) or DsRed2 vector (left) together with quantitative analysis (right panel).

Whether interaction between BRAP2 and the putative binding partners is functional in terms of altering subcellular localization was assessed in the presence of ectopically expressed DsRed2 fused to BRAP2 residues 343–592 (previously shown to be functional for BRAP2-NRNI activity^7^) in cells of either the COS-7, which endogenously express the T-ag, or HeLa cell lines. Effects were compared to those for DsRed2 expression alone, with the shorter BRAP2 construct, DsRed2-BRAP2 (442–592)^7^ also used. Cells were immunostained 20 h post transfection, using specific antibodies for endogenous PHLPP1 or DNMT1, or T-ag as a positive control in COS-7 cells, and Alexa 568-labeled secondary antibody, followed by imaging using CLSM. Like the positive control, PHLPP1 and DNMT1 showed reduced nuclear accumulation/increased cytoplasmic staining in presence of DsRed2-BRAP2 compared to in the presence of DsRed2 alone (Fig. 3b–d). Quantitative analysis confirmed these results, whereby determination of the nuclear to cytoplasmic fluorescence ratio (Fn/c) revealed that nuclear accumulation of PHLPP1 and DNMT1 was significantly reduced (p < 0.001; Fig. 3b, ci) in the presence of either DsRed2-BRAP2 construct, in comparable fashion to T-ag (p < 0.0001; Fig. 3d); DsRed2-BRAP2 (442-592) appeared to exert more extensive inhibition (50-70%), implying that BRAP2 residues 442-592 are sufficient for NRNI activity.

The effect of BRAP2 on localisation of DNMT1 was also confirmed in live cell experiments; where HeLa cells were cotransfected to express GFP fused full length human DNMT1 and DsRed2-BRAP2 (343-592) or DsRed2. GFP-DNMT1 showed almost 70% reduction in nuclear accumulation in the presence of Ds-Red2 BRAP2 (343-592 and 442-570), than in its absence (Fig. 3cii, p<0.0001). Overall, the results demonstrate that BRAP2 can function as an NRNI for PHLPP1 and DNMT1.

### PHLPP1, AKAP3 and DNMT1 are co-localised to the cytoplasm in germ cells expressing BRAP2 in mouse testis

**Figure 4:**
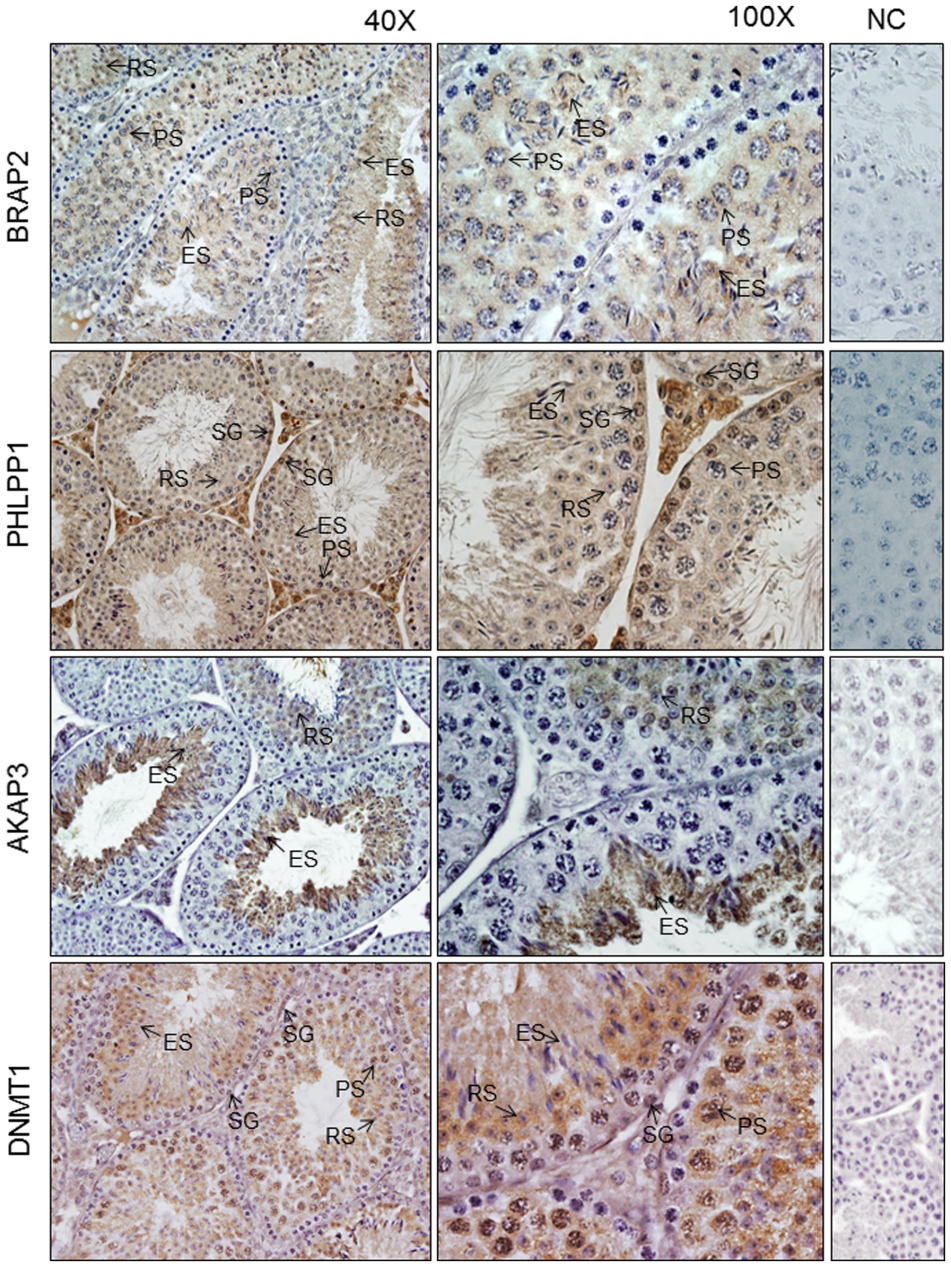
BRAP2 binding partners are localised to the cytoplasm in testicular germ cells co-expressing BRAP2. Immunohistochemistry of Bouins fixed paraffin embedded adult mouse testis sections from Asmu:Swiss mouse was performed for BRAP2, PHLPP1, AKAP3 and DNMT1. BRAP2 expression was observed in the cytoplasm of pachytene spermatocytes (PS), round (RS) and elongated spermatids (ES) as indicated (arrows). PHLPP1 was observed in nuclei of spermatogonia and is cytoplasmic in pachytene spermatocytes and round spermatids. AKAP3 was present in the cytoplasm of round and elongated spermatids. DNMT1 was present in the nuclei of spermatogonia and both in nucleus and cytoplasm of pachytene spermatocytes and only in cytoplasm of round and elongated spermatids. The panels at the right represents respective negative control showing testis sections with no primary antibody^30^.

To put the above results into a physiological context, expression and localisation of BRAP2, together with PHLPP1, AKAP3 and DNMT1 was examined using immunohistochemistry on adult mouse testis sections. Consistent with previous observations^14^, BRAP2 was detected in the cytoplasm of pachytene spermatocytes, round and elongated spermatids (Fig. 4). PHLPP1, in contrast, was nuclear in spermatogonia, but cytoplasmic in pachytene spermatocytes and round spermatids, the cell types coexpressing BRAP2, consistent with an NRNI role of BRAP2. DNMT1 was also nuclear in spermatogonia, but showed both cytoplasmic and nuclear expression in pachytene spermatocytes and was exclusively cytoplasmic in round spermatids, similarly consistent with cytoplasmic retention by BRAP2 in the cell types coexpressing it. Finally, AKAP3 was absent from earlier germ cell types, and present in the cytoplasm of round and elongated spermatids (Fig. 4), indicating that it is present in the same cell types as BRAP2. The results indicate clearly that in the meiotic and the post-meiotic germ cell types where BRAP2 is more highly expressed, all of its binding partners are strongly cytoplasmic, whereas in the premeiotic cell types with lower BRAP2 expression, PHLPP1 and DNMT1 are predominantly nuclear, implying that the NRNI activity of BRAP2 may play an important role during spermatogenesis.

### BRAP2 is present in sperm and colocalises with AKAP3

**Figure 5:**
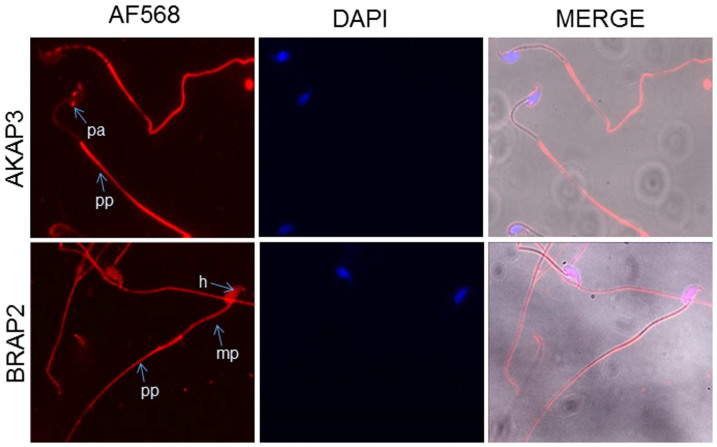
BRAP2 and AKAP3 colocalise in murine sperm. The localisation of BRAP2 and AKAP3 was examined in sperm collected from the epididymis of C57 Black adult wild type mice by indirect immunofluorescence microscopy using antibodies specific for BRAP2 and AKAP3 as per methods. Micrographs were taken ×100 magnification. h, sperm head, mp, mid piece; pp, principal piece; av, acrosomal vesicle; pa, post acrosomal region.

Immunofluorescence was also used to assess the localisation of BRAP2 in relation to that of AKAP3 in spermatozoa. Murine epididymal sperm were fixed and stained with anti-BRAP2, or -AKAP3 antibodies and Alexa 568-labeled secondary antibody, and examined by CLSM (Fig. 5). Results demonstrated that BRAP2 was expressed throughout the spermatozoa including the head, mid- and principle piece. AKAP3 was localised to the post acrosomal region and the principal piece demonstrating that BRAP2 and its newly identified binding partner are localised to the some of the same substructures of mature spermatozoa, consistent with the possibility of interaction of the two *in vivo*.

## Discussion

This is the first study to document the interactome of an NRNI from a tissue of physiological context, providing new information regarding BRAP2's role not only as an NRNI, but also in a more structural or scaffold role, including, potentially in mature spermatozoa. A number of novel putative binding partners for BRAP2 were identified, with roles in diverse cell processes, including cytoskeletal organisation, cell cycle/proliferation, gene regulation, and ubiquitinylation (see Table 1; Fig. 1b), implying a potentially important role of BRAP2 in these processes. We previously validated the high mobility group chromatin component HMG20A, the nuclear mitotic apparatus protein NUMA1 and nuclear envelope component SYNE2^14^ as binding partners of BRAP2; here we present validation of PHLPP1, DNMT1 and AKAP3 as novel BRAP2 interacting proteins in testis for the first time. In particular, immunoprecipitation from adult mouse testis lysates confirmed the ability of BRAP2 to bind the selected binding partners in the testis, while ectopic expression experiments demonstrated the ability of BRAP2 to act as an NRNI for PHLPP1 and DNMT1. Finally and most importantly, immunohistochemical staining in testis sections demonstrated that in the specific meiotic and post-meiotic germ cell types that express BRAP2, its binding partners PHLPP1, DNMT1 and AKAP3 are strongly cytoplasmic, consistent with an NRNI role for BRAP2 during spermatogenesis. Exactly how this relates to the specific function of the Akt regulator PHLPP1 or DNMT1, which maintains DNA methylation patterns in replicating cells, remains to be established, but since both modulate gene expression in different ways, it does not seem unreasonable to postulate that specific retention of these proteins in the cytoplasm by BRAP2 may be central to transcriptional control in the key cell types of pachytene spermatocytes and round spermatids.

**Figure 6:**
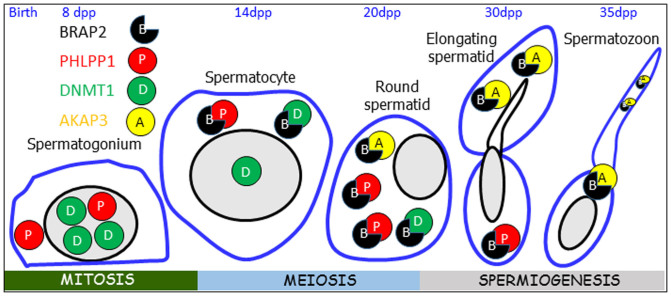
Postulated Model of BRAP2's role in spermatogenesis. In cell types such as the early spermatogonia where BRAP2 (blue) is expressed at low levels, BRAP2 binding partners such as PHLPP1 (red) and DNMT1 (green) are nuclear. Higher BRAP2 expression in the later germ cell types (pachytene spermatocytes, round and elongated spermatids), results in inhibition of nuclear targeting of PHLPP1 and DNMT1. BRAP2 protein persists late into spermiogenesis, being still present in the mature spermatozoan and hence able to interact with binding partners such as AKAP3 (yellow), which reach maximum levels in the mature spermatozoan. BRAP2 would appear to play a more structural/scaffold role to help localise proteins such as AKAP3 in the post acrosomal space and principal piece of sperm.

Intriguingly, we were able to show for the first time that BRAP2 is present in mature spermatozoa, in part colocalised with the testis specific protein, AKAP3 which targets protein kinase A to discrete locations within the cell, and thereby modulate sperm motility, capacitation, and the acrosome reaction^19^,^22^. Presumably BRAP2 may contribute to localising AKAP3 to specific areas of the mature sperm to help facilitate these functions; importantly, since AKAP3 is not implicated as having a nuclear role, it would appear that BRAP2's function in this context is not as an NRNI, but more as a scaffold or adaptor protein. That BRAP2 is localised all over the sperm may imply that its role in this context in the mature sperm may relate not only to AKAP3, but potentially to other proteins. A schematic model depicting the putative role of BRAP2 in spermatogenesis in modulating subcellular localisation is presented in Fig. 6.

In summary, this study reports the first tissue interactome for BRAP2. Importantly, it not only supports an important NRNI role for BRAP2 in meiotic and post meiotic germ cells in the testis in preventing nuclear access of proteins such as PHLPP1, DNMT1, HMG20A and NuMA1^19^,^22^, but also suggests a role for BRAP2 in the mature spermatozoan, potentially as a scaffold supporting the roles of proteins such as AKAP3. Detailed analysis of BRAP2 and its interactors is a high priority for future work in this laboratory, to continue to provide insight into BRAP2's role in spermatogenesis.

## Methods

### Ethics statement

The procedure of mouse tissue acquisition and experiments were performed in accordance with the guidelines of the NHMRC Code of Practice for the Care and Use of Animals for Experimental Purposes, and approved by the Monash University Standing Committee on Ethics in Animal Experimentation. The study was approved by the Monash University Research Committee.

### Yeast two-hybrid Screen

To identify BRAP2 binding proteins from testis, a high throughput Y2H system was performed in collaboration with Hybrigenics Inc. (Paris, France) of a random-primed human testis cDNA library using the C terminal fragment (343-592 amino acids) of human BRAP2 (gi: 188497704) as a bait.

### Collection of mouse tissues and epididymal sperm

Inbred C57BL/6J (B6) wild type male mice were obtained from Monash University Central Animal Services. Animals were culled by cervical dislocation prior to dissection of the organs and decapsulation (testis and kidneys). To collect sperm the epididymis was nicked and incubated in PBS + 5% FCS for 15–25 min at 37°C. Briefly, sperm released from epididymis were washed thrice using PBS for 5 min at 300 × *g*, resuspended in PBS, and air dried onto polylysine-coated slides as described previously^23^,^24^.

### Expression plasmid construction

DsRed2-tagged BRAP2 mammalian expression vectors were constructed by inserting the sequences encoding BRAP2 amino acids 343–592 and 442-592, amplified by polymerase chain reaction (all primer sequences used are listed in supplementary Table 1) and cloned into the *HindIII* and *BamHI* restriction endonuclease sites, C-terminal to the coding sequence of DsRed2, of plasmid DsRed2-C1 (Clontech, Mountain View, CA, USA). The GFP-fused BRAP2 (encoding amino acids 343–592) expression vector was constructed as described previously^7^, using the Gateway system (Invitrogen, Carlsbad, CA, USA); in the plasmid vector pDONR207. LR recombination reactions were subsequently performed, using the Gateway-compatible destination vector pEPI-GFP^25^ to generate GFP-fusion protein-encoding constructs for mammalian cell expression. The integrity of all constructs was verified by DNA sequencing. The mammalian expression plasmids encoding GFP-DNMT1^26^ and GST-AKAP3^19^ were generous gifts from Dr. Heinrich Leonhardt (Faculty of Biology at the LMU, Munich) and Dr. Daniel W. Carr (School of Medicine, Endocrinology; Oregon health and Science universities, Portland), respectively. Plasmids pcDNA3/Myc-DNMT1^27^, pcDNA3-HA-PHLPP1α^18^, and pcDNA3 HA-PHLPP1β^28^ were from Addgene (numbers 36939, 22404 and 37100 respectively).

### Cell culture

African green monkey kidney COS-7, human cervical cancer HeLa and human embryonic kidney HeK 293T cell lines were cultured and maintained in DMEM/EMEM containing 10% fetal calf serum in a 5% CO_2_ humidified incubator at 37°C^7^.

### Transfection/immunofluorescence

Cells seeded onto glass coverslips were transfected using Lipofectamine® 2000 (Invitrogen) according to the manufacturer's specifications. 20–24 h post transfection, cells were fixed^7^ and incubated in anti-SV40 T-ag (Santa Cruz, 1:800) or anti-PHLPP1 (Novus Biologicals, 1:200) or anti-DNMT1 (Abcam, 1:200) primary antibodies, followed by incubation with Alexa 568-labeled goat anti-rabbit secondary antibody or Alexa 568-labeled rabbit anti-mouse secondary antibody (Invitrogen, 1:1000). DNA was counterstained with DNA-specific dye 4,6-diamidino-2-phenylindole (DAPI) using ProLong® Gold Anti-Fade Reagent (Invitrogen).

Immunofluoresence on epididymal sperm was performed using a protocol modified from Herrero et al., 1996^23^. Sperm were fixed with 4% PFA for 10 mins, permeabilized with Triton X-100 (0.1%) and blocked with 1% BSA. Sperm were incubated with anti-BRAP2 (1:200) or anti-AKAP3 (1:100) primary antibody at 4°C overnight, followed by Alexa 568-labeled goat anti-rabbit secondary antibody (Invitrogen, 1:1000) and mounted using ProLong® Gold Antifade with DAPI (Invitrogen Laboratories). Images were captured using a FluoView™ FV1000 confocal microscope, using 100X oil immersion objective lens.

### CLSM and image analysis

Cells immunostained for endogenous proteins or transfected to express DsRed2 fusion proteins were imaged using a FluoView™ FV1000 Confocal Microscope equipped with 100x oil immersion objective lens. For assessing subcellular localization of GFP and DsRed2 fusion proteins in live cells; CLSM analysis was done similarly on a FluoViewTM FV1000 confocal microscope equipped with a FCS2 live-cell chamber and temperature controller maintained at 37°C (Bioptechs, Butler, PA, USA). The nuclear to cytoplasmic fluorescence ratio (Fn/c) was determined as previously described^7^ from digitized images using the ImageJ 1.43r public domain software (NIH), statistical analysis performed using a 2-tailed unpaired t-test and the Microsoft excel software.

### Lysate preparation/coimmunoprecipitation/Western analysis

Adult mouse tissues were dissected from C57 black mice, as per Section 2.2. Following PBS washes, tissues were homogenized using RIPA buffer (150 mM sodium chloride, 1% NP40, 0.5% sodium deoxycholate, 0.1% SDS, 50 mM Tris, pH 8) containing a protease inhibitor cocktail (Roche), cell pellets were lysed using a lysis buffer as described previously^7^. Cellular debris was removed by centrifugation at 20,000 × *g* for 30 min at 4°C. Protein concentration of the cleared lysate was determined by Bradford assay (Biorad).

Co-immunoprecipitation was performed from 400 μg of mouse testis lysate using the Catch and Release® v2.0 Reversible Immunoprecipitation System (Upstate Cell Signalling Solutions) according to the manufacturer's instructions, using 4 μg of anti-PHLPP1 (Novus Biologicals), anti-AKAP3 (Protein tech), anti-DNMT1 (Abcam), anti-BRAP2 (Sigma), or anti-GST (Santa Cruz) antibodies. Following overnight incubation, proteins were eluted from the beads with successive 60 μl additions of 1 ×, 2 × and 4 × non-denaturing buffer and 1 × denaturing buffer, and subjected to SDS-polyacrylamide gel electrophoresis (10% gel, or 8%).

To pull down GFP fusion proteins, cell lysates were incubated with GFP-trap beads (Chromotek) per manufacturer's instructions, for 1 h at 4°C with gentle rotation. The beads were then washed three times with GFP-trap wash buffer and resuspended in SDS–PAGE sample buffer. For detection of GFP fusion proteins by immunoprecipitation, 30 μl of the eluate was subjected to SDS-polyacrylamide gel electrophoresis (10% gel).

After electrophoresis, proteins were transferred to Polyvinylidene Fluoride (PVDF) membranes (PALL Corporation) preactivated in isopropanol (Merck) and Western blotting carried out as previously described^7^. Briefly, the membrane was blocked in 5% skim milk powder in PBS and incubated overnight at 4°C with rabbit anti-BRAP2 (1:1000), anti-PHLPP1 (1:750 or 1:1000, as appropriate), or mouse anti-DNMT1 (1:1000) primary antibodies. The anti-HA antibody (Cell Signalling) was used for detection of HA-PHLPP1 proteins; anti-myc antibody (Santa Cruz) for Myc-DNMT1 and anti-GST antibody for detection of AKAP3-GST proteins. Subsequently, blots were incubated in HRP-coupled goat anti-rabbit or goat anti-mouse secondary antibody (Millipore, 1:10,000), for 1 h and detected using enhanced chemiluminescence (ECL) (Perkin Elmer) according to the manufacturer's instructions. Tissue and cell lysates (25 μg) were subjected to SDS-polyacrylamide gel electrophoresis (10% gel) and Western transfer, blocking, incubation with anti-BRAP2 primary antibody and ECL detection performed essentially as above.

### Immunohistochemistry

Immunohistochemistry was performed on Bouins-fixed paraffin-embedded adult Asmu:Swiss mouse testes sections using an avidin-biotin amplified immunohistochemical method as described previously^29^. The testis sections were incubated with primary antibody diluted in 0.5% BSA in TBS overnight in a humid chamber at 4°C {anti-BRAP2 (1:500), anti PHLPP1 (1:100); anti-AKAP3 (1:200) or anti-DNMT1 (1:400)}. Bound primary antibody was detected using biotinylated anti-rabbit/anti-mouse antibody (Millipore) diluted 1:500 in 0.5% bovine serum albumin (BSA)/PBS. Negative controls received 0.5% BSA in TBS in place of primary antibody. The sections were incubated with Vectastain® (Vector laboratories) washed and counterstained with Harris' Hematoxylin (BioRad), and mounted onto coverslips using DPX (di-n-butyl-phthalate in xylene) mounting solution (Sigma) and imaged using a bright field microscope (Provis) with 40 or 100 × oil immersion lens.

## Supplementary Material

Supplementary Information Supplementary Table 1

## Figures and Tables

**Table 1:** List of BRAP2 interacting proteins identified in this study. A Y2H screen was performed on a human testis cDNA library using human BRAP2 (343–592) as a bait to identify BRAP2 interactors. Predicted biological score (PBS) indicates confidence of interaction (A is highest); selective interactive domain (SID) is the minimal sequence shared by all fragments for the binding partner identified that interact with BRAP2

BRAP2 Interacting Protein (Protein Accession Number)	PBS	SID	Subcellular localisation: Cellular role
1. EFHC1, EF-hand domain (C-terminal) containing 1 (NP_060570.2)	A	372-584	Cytoplasmic/cell membrane; associates with mitotic spindle, may enhance calcium influx and stimulate apoptosis
2. UBB, Ubiquitin B (NP_061828.1)	A	71-91	Cytoplasmic: role in proteosomal degradation, DNA repair, cell cycle, apoptosis
3. UBC, Ubiquitin C (NP_066289.2)	A	1-137/309-321/452-475	Cytoplasmic: role in proteosomal degradation
4. APOA1, Apolipoprotein A-1 (NP_000030.1)	B	85-228	Extracellular space: lipid transporter that binds to high-density lipoprotein
5. NUMA1, Nuclear mitotic apparatus 1 (NP_006176.2)	B	857-923	Cytoplasmic/nuclear/spindle microtubule/spindle pole: role in cell cycle, interacts with mitotic spindle^14^
6. SYNE2, Spectrin repeat containing, nuclear envelope 2 (NP_878918.2)	B	5986-6126	Cytoplasm, membrane/interacts with actin, cytoskeletal anchoring at nuclear membrane^14^
7. C3ORF10, Chromosome 3 open reading frame 10/BRICK1 SCAR (AAN60161.1)	C	24-75	Cytoplasm and microtubule cytoskeleton/role in regulation of actin cytoskeleton
8. CCDC105, Coiled-coil domain containing 105 (NP_775753.2)	C	181-348	Nucleus, microtubule cytoskeleton/role in microtubule cytoskeleton organization
9. C1ORF14, Chromosome 1 Open Reading Frame 14 (NP_112195.2)	D	51-387	Cytoplasmic
10. C1ORF49, Chromosome 1 Open Reading Frame 49 (NP_115502.1)	D	19-233	Microtubule cytoskeleton/nuclear membrane/nuclear
11. DNMT1, DNA (cytosine-5-)-methyltransferase 1 (NP_001370.1)	D	699-833	Nuclear/cytoplasmic: DNA (cytosine-5-)-methyltransferase with role in chromatin modification/cell proliferation
12. PHLPP1, PH domain and leucine rich repeat protein phosphatase 1 (NP_919431.1)	D	1-137	Cytoplasmic/nuclear/membrane: protein serine/threonine phosphatase activity inhibitor of k-Ras signalling, promotes cell apoptosis
13. FAM184A, Family With Sequence Similarity 184, Member A (NP_001093881.1)	D	483-612	Nuclear
14. FBX02, F-Box Protein 28 (NP_055991.1)	D	51-338	Cytoplasmic: probably binds to some phosphorylated proteins and promotes their ubiquitination and degradation
15. C3ORF42, Chromosome 5 Open Reading Frame 42 (NP_075561.2)	D	2000-2043	Cytoplasmic/cell membrane
16. ZNF382, Zinc Finger Protein 382 (NP_116214.2)	D	161-365	Nuclear: transcriptional repressor
17. ZNF479, Zinc Finger Protein 479 (NP_150376.1)	D	248-458	Unknown
18. AKAP3, A kinase (PRKA) anchor protein 3 (NP_006413.2)	D	772-853	Acrosomal vesicle/sperm flagellum: scaffold for protein kinase A with roles in the acrosome reaction/sperm motility
19. BRAP2, BRCA1 associated protein (NP_006759.3)	D	107-350/454-592	Cytoplasmic: negative regulator of nuclear import
20. TANC2, Tetratricopeptide repeat, ankyrin repeat and coiled-coil containing 2 (NP_079461.2)	D	1257-1450	Extracellular space: role in embryonic development
21. RBM12, RNA binding motif protein 12 (NP_690051.1)	D	826-924	Cytoplasmic/nuclear/mitochondrion: binds to RNA, may play a role in RNA processing and modification.
22. ZNF822, Zinc Finger Protein 822 (NP_056066.2)	D	59-206	Nuclear/cytoplasmic: role in response to DNA damage and apoptosis
23. CCDC30, Coiled-coil domain-containing protein 30 (NP_001074319.1)	D	448-783	Cytoplasmic/nuclear
24. SMARCE1, SWI/SNF related, matrix associated, actin dependent regulator of chromatin, subfamily e, member 1 (NP_003070.3)	E	36-277	Mitochondrion/nuclear: transcriptional repressor
25. ZNF521, Zinc finger protein 521 (NP_056276.1)	E	44-336	Nuclear: transcriptional activator or repressor
26. HMG20A, High mobility group 20A (AAH21959.1)	E	6-314	Nuclear: role in neuronal differentiation^14^
