# Supplementary material for: Interactome of the negative regulator of nuclear import BRCA1-binding protein 2
Source: Sci Rep. 2015 Mar 30;5:9459. doi: 10.1038/srep09459 (PMC4377634; doi:10.1038/srep09459)
Supplement: Supplementary Information — Supplementary Table 1 [file srep09459-s1.doc]

**Interactome of the negative regulator of nuclear import BRCA1-binding protein 2**

Shadma Fatima^1^, Kylie M. Wagstaff^1^, Kate L. Loveland^1, 2^ and David A. Jans^1*^.

^1^Department.of Biochemistry & Molecular Biology Monash University, Clayton, Victoria, Australia

^2^Department of Anatomy and Developmental Biology, Monash University, Clayton, Victoria, Australia

Supplementary Table 1. Oligonucleoltide primers used for PCR in this study.

| **Construct name** | **Primer name** | **Sequence** |
| --- | --- | --- |
| DsRed2-BRAP2-343-592 | Fwd- Dsred-BRAP-343 (HindIII) | 5’-CGCAAGCTTCCCGACATGCTTATAAGCAC-3’ |
|  | Rev- Dsred-BRAP-592 (BamHI) | 5’-CGC GGA TCC TTA CTT GCC CCT CTT-3’ |
| DsRed2-BRAP2-442-592 | Fwd- Dsred-BRAP-442 (HindIII) | 5’-CGCAAGCTTCCAAGACCAAGTTTAAAGAAAC-3’ |
|  | Rev- Dsred-BRAP-592 (BamHI) | 5’-CGC GGA TCC TTA CTT GCC CCT CTT-3’ |
| GFP-BRAP2-343-592 | Fwd- attB1-BRAP2-343 | 5’GGGGACAAGTTTGTACAAAAAAGCAGGCTTACGACATGCTTATAAGCACTTTGAGG-3’ |
|  | Rev- attB2-BRAP2-592 | 5’-GGGGACCACTTTGTACAAGAAAGCTGGGTCACTTGCCCCTCTTGCTGCGG-3’ |
